# Climate control on the channel morphodynamics of the Sittaung River, Myanmar

**DOI:** 10.1038/s41598-024-58198-1

**Published:** 2024-03-29

**Authors:** Luke Stefan Bisson, Kyungsik Choi

**Affiliations:** School of Earth and Environmental Sciences and Research Institute of Oceanography, Seoul National University, Seoul, Republic of Korea

**Keywords:** Geomorphology, Environmental sciences, Solid Earth sciences

## Abstract

The spatio–temporal development of a meandering river is controlled by its channel morphodynamics. In regions of rapid channel evolution, understanding the driving factors of meander migration is crucial in forecasting the rate and extent of morphological change. Sediment supply and fluvial discharge are the primary influences on migration rate, however climate oscillations are also integral in indirectly regulating migration rate through their control of regional precipitation, as well as the monsoon season of sub-tropical Asia. Despite this, an in-depth investigation into the impact of climate oscillations on meander bend migration remains undocumented. This study presents a satellite-based analysis of multi-decadal climatic forcing on the migration rate of the Sittaung River in Myanmar, through interpretation of the El Niño Southern Oscillation (ENSO). The mode of ENSO exerts significant climatic control on the migration rate of the meandering channels of the Sittaung River, with low-to-average migration rates recorded during dry El Niño events and peak migration rates observed during wet La Niña events. However, this climatic signal may have been obscured by certain local environmental conditions. In cases where meanders faced geological basement, the basement rock inhibited their migration through extension, forcing more rapid migration by way of seaward translation. Consequently, these translating meanders developed to be more elongate, with lower curvatures. Meanders downstream of the approximate tidal limit were less downstream skewed, indicative of tidal modulation, potentially obscuring the impact of fluvially driven climate forcing. Additionally, downstream of a major confluence, the input of sediment and fluvial discharge may have been regulated by upstream anthropogenic activities such as mining and dam construction, leading to greater variability in migration rate downstream of this confluence and further obfuscation of the climate signal.

## Introduction

The channel morphodynamics of fluvial and tidal meanders are driven by a complex combination of climatic, terrestrial, and anthropogenic factors. Understanding channel morphodynamics not only aids our interpretation of the sediment record^1^, but also has many practical applications. Quantifying the extent of fluvial and tidal modulation of a river channel is important in the development of fluvial models and flood mitigation^2,3^, as well as in impact assessments on the hydropower and agricultural sectors^4^. This study presents an in-depth analysis of how meander bend migration rate is influenced by climate forcing, while also investigating the local morphodynamic, environmental, and anthropogenic conditions which may obscure a potential climate signal.

The rate of migration of any given meandering channel is inherently governed by its morphodynamics. A linear relationship exists between curvature and migration rate where, beyond the point of peak curvature, migration rate decreases with increasing curvature^5,6^. However, this long-held assertion has recently been challenged^7^. The mode of meander migration also exerts an influence on migration rate, with meanders predominantly migrating through translation exhibiting faster migration rates than those migrating through extension^8^. Bend asymmetry changes throughout the life of a natural meander bend, with meanders beginning with a downstream-skew and developing into an upstream-skew with increasing sinuosity^9^. The sinuosity of meandering river channels is governed by the slope of the channel, its flow resistance, as well as the extent of bank vegetation^10^. Vegetation brings stability to meander bend sediments, hindering migration^11^. Likewise, meander bends entrenched in bedrock also exhibit greater stability and lower rates of migration^12^. Flow convergence and scour holes present at confluences introduce further complexities into the study of confluent meander bends, at which channel morphodynamics and flow regimes can differ greatly from regular, non-confluent bends^13,14^. In the case of sediment rich tributaries, deposition downstream of confluences has been shown to increase as load increases^15^. The supply of sediment to a meandering channel may aid or hinder its migration^6^. An observable example of this is in the event of chute cut-offs, where the fluvial regime rapidly becomes analogous to that of a confluence^16^, often resulting in a substantial influx of sediment into channels, causing accelerated evolution in their morphology^17^.

The aforementioned morphodynamics of a channel as well as its long-term evolution are driven by its fluvial discharge regime, with its erosional and depositional capacity controlled to an extent by climatic forcing, most notably by precipitation^18^. Meander migration rate is known to be highly sensitive to fluvial discharge, flood events, and suspended sediment concentration^19,20^. Towards the river mouth, tidal processes have been shown to modulate fluvial discharge, influencing the transport and deposition of sediment^21–23^. The seasonal precipitation regime of a region controls the morphodynamics of its river channels and their sedimentary facies’ development, particularly in areas influenced by monsoon rains^24^. Monsoon rains drive fluvial discharge, accelerating erosion and suspended sediment transport^25–27^. Climate oscillations such as the El Niño Southern Oscillation (ENSO), North Atlantic Oscillation (NAO), and Pacific Decadal Oscillation (PDO) are macroscale atmospheric patterns, with the capability to influence regional weather patterns, impacting precipitation, fluvial discharge, and flood intensity^28,29^; thus, they may indirectly force or limit the migration of fluvial river channels.

The climate of sub-tropical Asia is governed by the position and intensity of the summertime monsoon^25–27^. The intensity of El Niño and La Niña events has been shown to influence the position of the Walker circulation^30^, driving both the location and magnitude of monsoon precipitation. The regional climate of Myanmar is sensitive to the phase of a number of such teleconnections. From May to November, both the ENSO and PDO influence the summer monsoon in the region^31^. La Niña events drive periods of intense precipitation^32^, while El Niño events and the positive mode of the Indian Ocean Dipole (IOD) lead to high temperatures and periods of drought^33^. The positive phase of the PDO drives higher precipitation, while the negative phase conversely leads to drier conditions^34^. Significantly, during years devoid of a strong ENSO signal, the effect of the PDO on the summertime monsoon has been shown to be influential^35^. Other climate oscillations such as the Boreal Summer Intraseasonal Oscillation (BSISO), as well as the orography of a region can also influence the extent of these monsoon rains in Myanmar^36^.

The Sittaung River Basin in Myanmar has an at-risk population living on its active floodplains^37,38^; understanding the multi-decadal migration of the river is of vital importance to their lives and livelihoods. Intensive tin, gold, and jade mining activities across the country affect the sediment flux of the Sittaung^39–42^, while upstream damming can cause unnatural variation in its fluvial discharge regime and reduction in peak flood discharge^43–47^. The annual migration of the Sittaung’s channels is visible from satellite imagery. Its rapid migration makes the Sittaung an optimal location for the study of channel morphodynamic sensitivity to annual climate fluctuations, yet research into the river’s sensitivity to climate forcing is as of yet incomplete^4^. There is a lack of observational data available^4^, thus the Sittaung is an ideal case for a satellite-based approach.

The factors directly influencing channel morphodynamics have been studied in detail, yet presently there is insufficient documentation relating to indirect forcing controlled by climate oscillations. By understanding the indirect influence of climate on channel morphodynamics, multi-decadal patterns of channel migration can be interpreted and analysed with greater accuracy. This study aims to quantify the influence of major climate oscillations on the morphodynamics of the Sittaung River, through analysis of the time series of meander bend migration for a series of 31 meander bends over a 35-year period from 1988 to 2023.

## Study area

**Figure 1:**
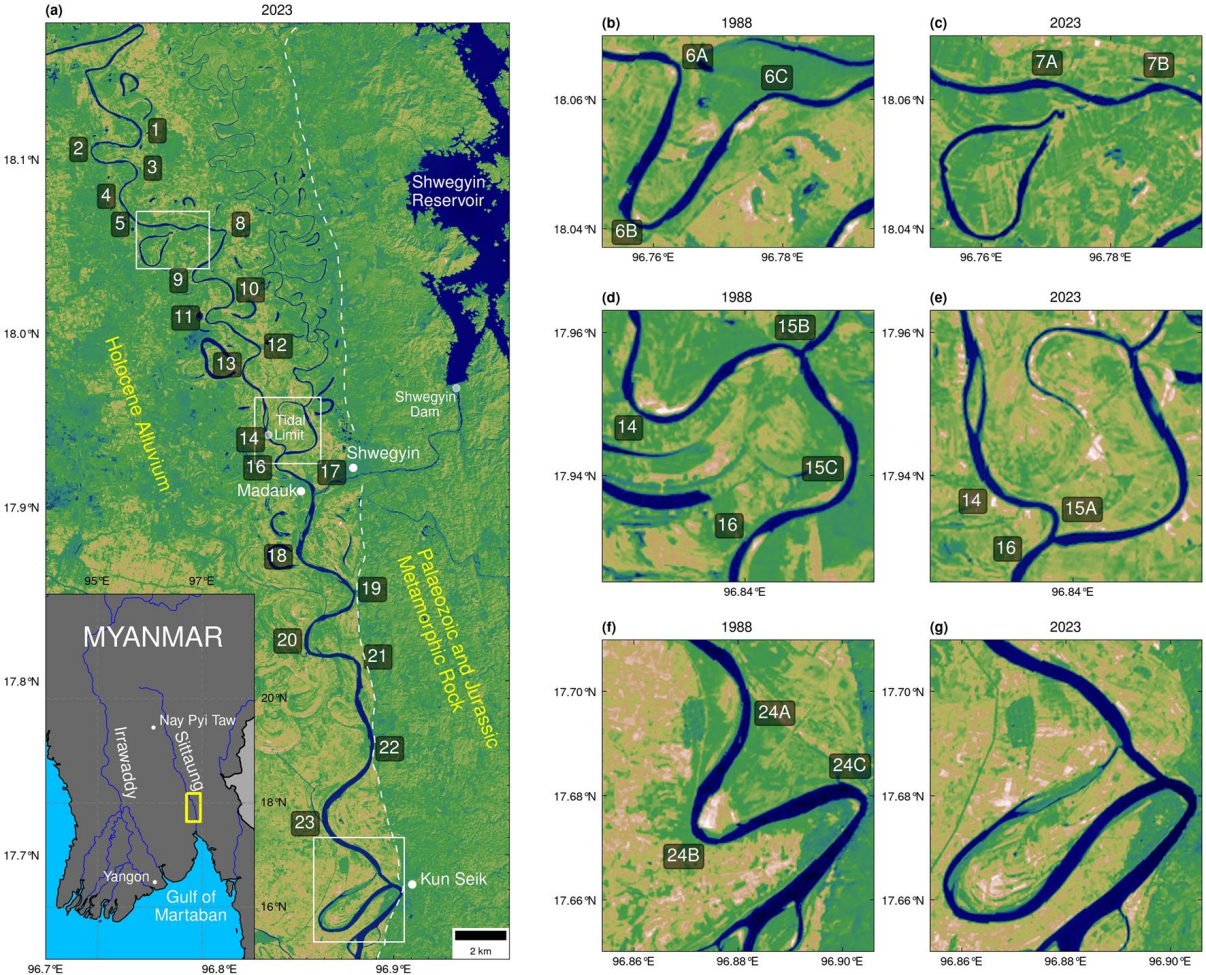
Maps showing the location of (**a**) the study area, (**b**, **c**) cut-off between meanders 6A and 6C, (**d**, **e**) cut-off between meanders 14 and 16, and (**f**, **g**) cut-off between meanders 24A and 24C. The white dashed line delineates the boundary between Palaeozoic and Jurassic metamorphic rock to the east and Holocene alluvium to the west. Landsat images courtesy of the U.S. Geological Survey.

The Sittaung River lies east of the Irrawaddy Delta in Myanmar, running approximately 420 km from its source to its mouth in the Gulf of Martaban. The river flows through an alluvial valley, alongside the Shan Scarp fault, flanked by the Pegu range to the west and the Shan Highlands to the east. From source to mouth, the majority of the Sittaung lies upon a large sedimentary basin, however its southern stretch is flanked by metamorphic basement rock along its eastern side^48^ (Fig. 1a).

The study area comprises a downstream stretch of the Sittaung (Figs. 1a, S1), lying between 75 and 130 km from the river mouth^49^. Here, the river is fluvially dominant, yet tidal influence^49^ exists during the dry season. This particular stretch of the Sittaung is influenced by a wide variety of local environmental factors. As such, each meander bend within the study area presents a unique case of channel morphodynamic conditions.

The Sittaung has dynamic and rapidly changing channels^50^, with minimal anthropogenic impacts from channel engineering. There is a lack of heavy bank vegetation or dense urbanisation along the studied stretch of meanders which could influence their rate of migration. Only a few major settlements lie across the study area: Madauk to the west, Shwegyin to the east, and Kun Seik to the south (Fig. 1a). Despite this, the construction of the Shwegyin Dam and reservoir (Fig. 1a) and the initiation of mining activities^39^ to the northeast of the study area are likely to have led to the input of sediment at the confluence near Shwegyin, and increased turbidity along the downstream stretch of meanders.

There exists a great degree of seasonality in the precipitation and discharge regimes of the Sittaung, controlled by the extent of monsoon rainfall during the months of May to October^32^. In addition to climatic and fluvial controls on the Sittaung’s channel morphodynamics, the evolution of its channel morphology downstream of the approximate tidal limit at Nyaung Bin Bak^49^ (Fig. 1a) is increasingly influenced by tidal processes^49,50^. Monthly, annual, decadal, multi-decadal, and centennial cycles of channel migration have been proposed for the tidally influenced stretch of the Sittaung^50,51^, but there has not yet been a comprehensive comparison between these tidally modulated channels and the solely fluvial portion of the river further upstream.

## Methodology

### Data

Landsat-5, 7, 8, and 9 imagery is used in this study (Landsat images courtesy of the U.S. Geological Survey). Centrelines were generated from band 4 and band 5 Landsat data, given its effectiveness in defining channel banks. The Sittaung River is covered by cloud during Myanmar’s extensive monsoon season; as such, clear Landsat images were selected from the winter months of each year. In spite of this, difficulties existed in the image selection process due to the presence of clouds and data-gaps existing in Landsat-7 images after 2003^52^.

This study uses the Total Precipitation and Mean Run-off Rate variables of the European Centre for Medium-Range Weather Forecasts (ECMWF) Reanalysis v5 (ERA5) “ERA5 monthly averaged data on single levels from 1940 to present” dataset^53^, obtained from the Copernicus programme, to identify spatial and temporal trends in precipitation and run-off rate across the Sittaung River drainage basin. In the absence of a reliable time series of fluvial discharge data, these variables are used as proxies to quantify the influence of climate oscillations on the fluvial discharge of the Sittaung River. The resolution of this longitude-latitude gridded dataset is approximately 30 km by 30 km.

The Niño 3.4 Index was used to interpret the signal of ENSO within the migration rate time series. It represents the monthly anomaly of averaged Sea Surface Temperature (SST) in the mid-Pacific and is currently the official SST Index provided by the National Oceanic and Atmospheric Administration (NOAA) for use in ENSO monitoring and analysis. A summertime average anomaly of the months of June, July, and August was used to identify summertime monsoon forcing. The ENSO, PDO, and IOD indices used in this study were obtained from the NOAA Physical Sciences Laboratory (PSL) (data provided by the NOAA PSL, Boulder, Colorado, USA, from their website at https://psl.noaa.gov/).

### Methods

Traditionally, studies have calculated meander migration rate from georeferenced aerial photography or LiDAR imagery^6,54^, or through masking the channel^55^. This study applies an alternative methodology comprising the automated generation of channel centrelines from Landsat data. Prior studies have primarily utilised Otsu’s method of thresholding^56^ to define channel banks^57–60^, however local variabilities may result in segmentation limitations when applying a global threshold across the entire stretch of a river. As such, this study applies an adaptive thresholding method as part of the centreline generation process.

All processes, from the input of Landsat data, to the output of meander bend vector point data, were performed within the QGIS program^61^. The Landsat data was passed through a thresholding algorithm and skeletonised to produce channel centrelines. The resultant centrelines were smoothed and split to create 100 equidistant points along each meander bend. Supplementary Fig. S2 displays a flowchart outlining the centreline generation procedures performed in QGIS.

Directional bearings were calculated at each data point along the stretch of the centreline, and used to identify the beginning and end of each meander bend^62,63^. The start of each meander bend was identified where there was a continuous positive or negative trend in these directional bearings. The end of each meander bend was identified when this continuous trend subsided. In some cases, between identified meander bends, straight stretches of the centreline existed where the directional bearings showed no continuous trend, making it difficult to attribute these stretches to either meander. Only clearly defined meander bends were selected in order to limit misinterpretation of the results calculated from the bend co-ordinate data^54^.

### Calculations

The calculations of displacement, migration rate, and curvature used in this study are based upon the formulae described in Marani et al.^64^ and Finotello et al.^54^.

1 $$\delta_{n} = \sqrt {\left[ {x_{n} \left( {t + {\Delta }t} \right) - x_{n} \left( t \right)} \right]^{2} + \left[ {y_{n} \left( {t + {\Delta }t} \right) - y_{n} \left( t \right)} \right]^{2} }$$

Displacement (δ_*n*_) of any given point *n* along the centreline of a meander bend is calculated with reference to its Cartesian co-ordinates, *x* and *y*^54,64^. δ_*n*_ is calculated for each of the 100 equidistant data points along the meander bend at the start (*t*) and end (*t* + Δ*t*) of the selected time period.

2 $$\zeta_{n} = \delta_{n} /\left( {{\Delta }t} \right)$$

**Figure 2:**
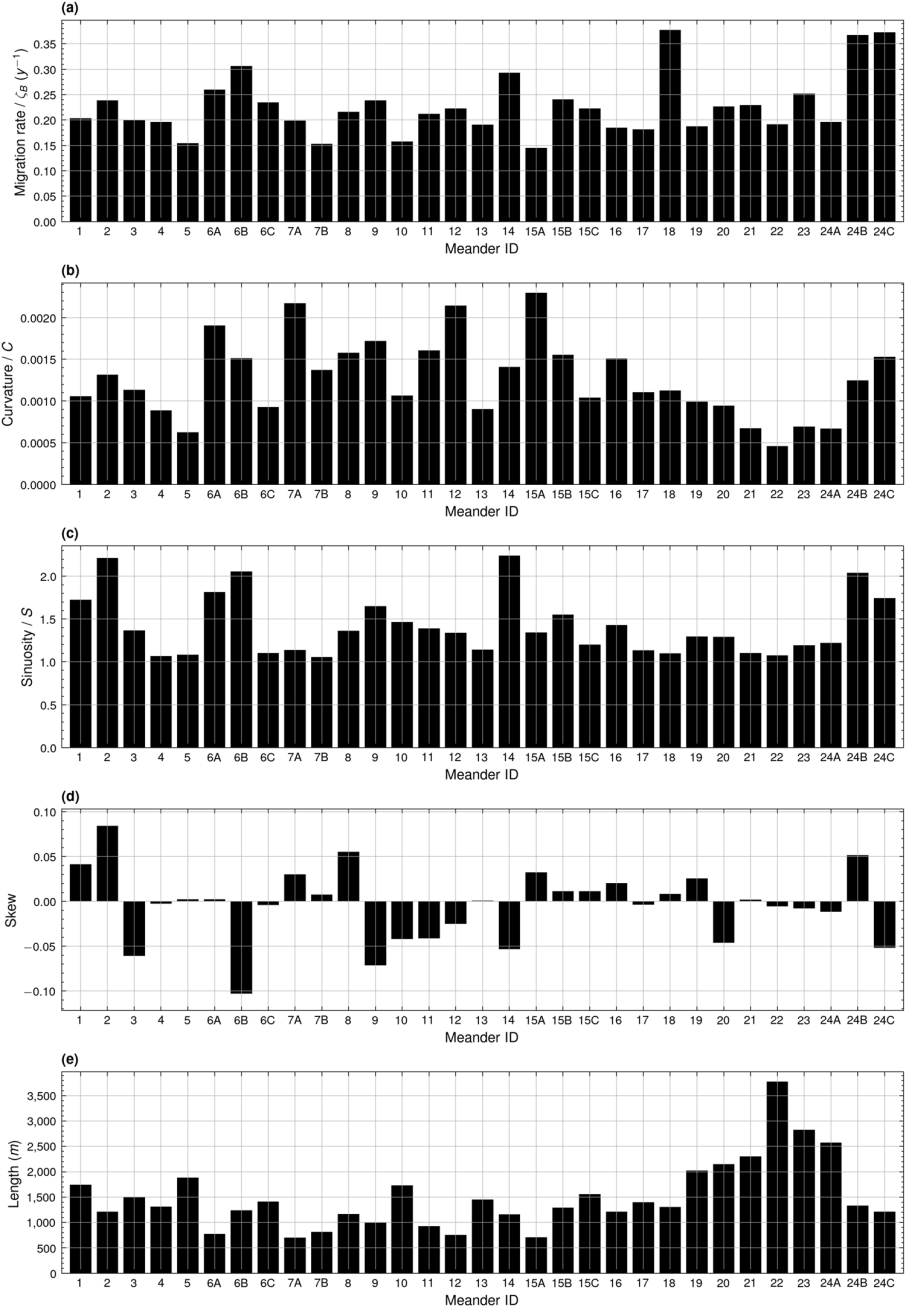
Bar graphs showing the average alongstream meander bend (**a**) migration rate (y^−1^), (**b**) curvature, (**c**) sinuosity, (**d**) skewness, and (**e**) bend half-wavelength (m) for the years of 1988 to 2023.

**Figure 3:**
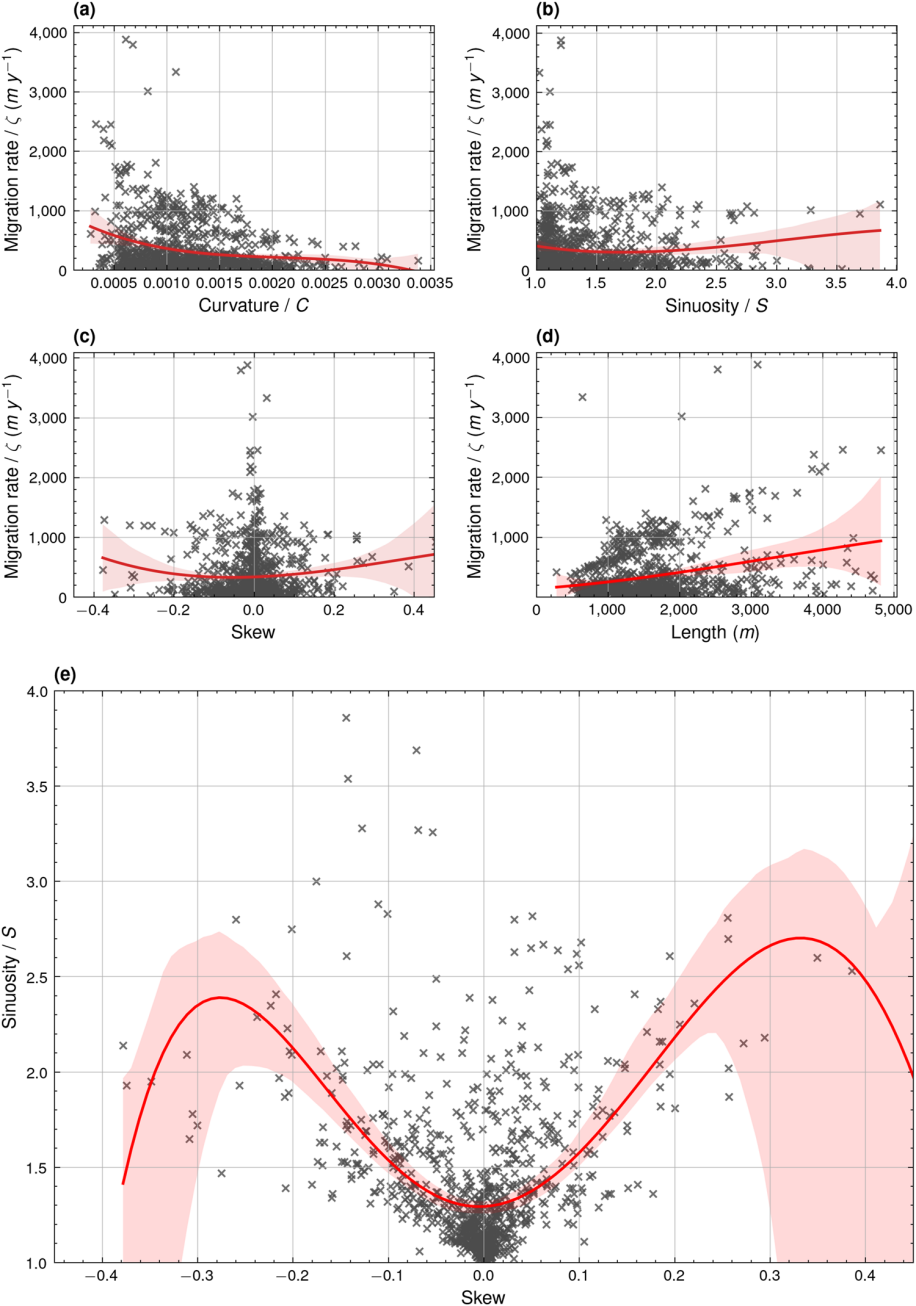
Third-order regression plots and 95% confidence intervals showing the relationships between meander bend (**a**) migration rate (m y^−1^) and curvature (R^2^: 0.06, F: 20.77, *p* value: 4.96e^−13^, df: 926), (**b**) migration rate (m y^−1^) and sinuosity (R^2^: 0.01, F: 2.39, *p* value: 0.07, df: 926), (**c**) migration rate (m y^−1^) and skewness (R^2^: 0.01, F: 2.20, *p* value: 0.09, df: 926), (**d**) migration rate (m y^−1^) and bend half-wavelength (m) (R^2^: 0.09, F: 28.65, *p* value: 1.01e^−17^, df: 926), and (**e**) fifth-order regression plot and 95% confidence intervals showing the relationship between sinuosity and skewness (R^2^: 0.32, F: 87.08, *p* value: 4.70e^−75^, df: 924).

**Figure 4:**
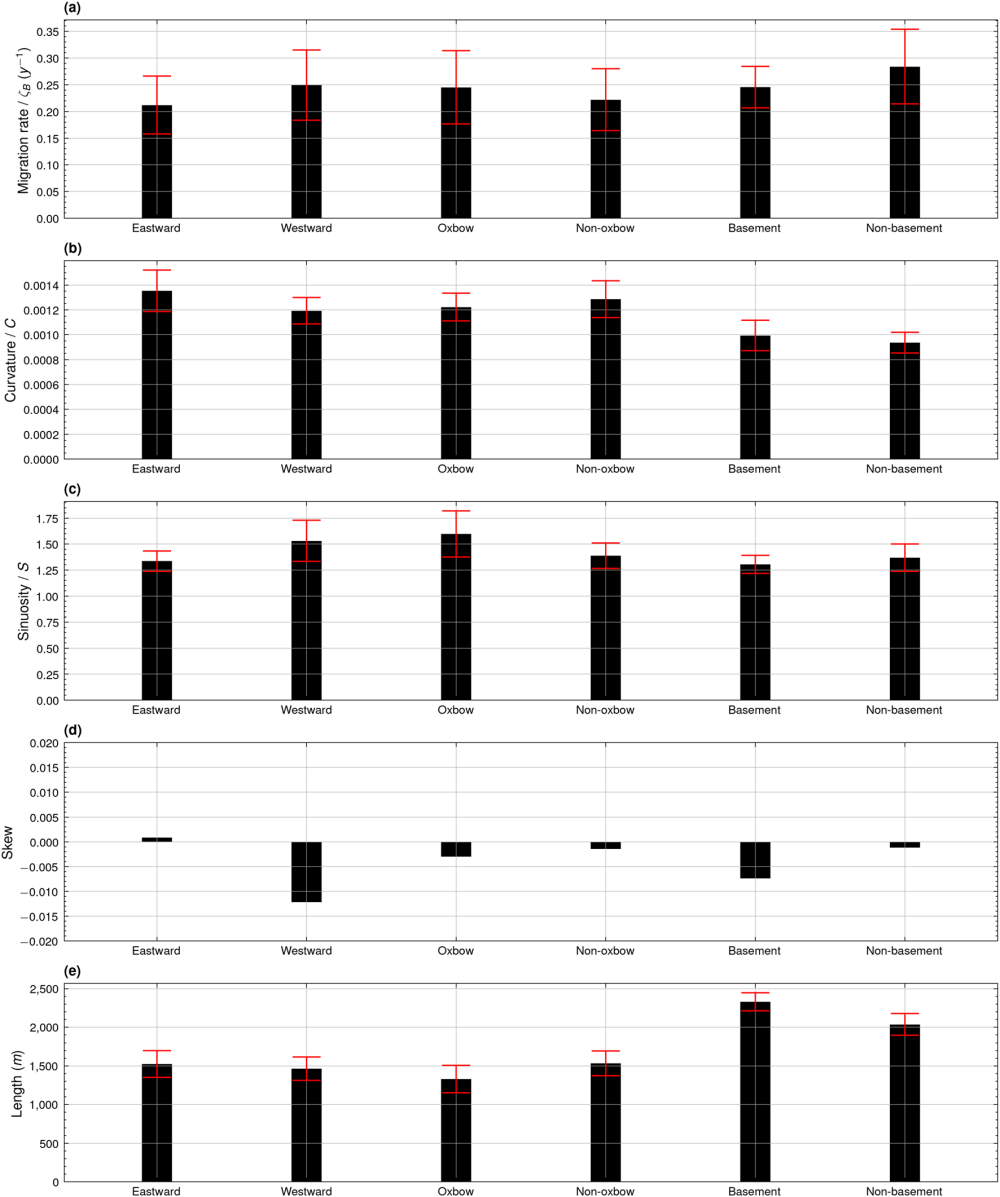
Bar graphs showing average alongstream meander bend (**a**) migration rate (y^−1^), (**b**) curvature, (**c**) sinuosity, (**d**) skewness, and (**e**) bend half-wavelength (m) of eastward facing meanders (Eastward), westward facing meanders (Westward), meanders with proximal oxbow lakes (Oxbow), meanders without proximal oxbow lakes (Non-oxbow), downstream meanders bounded by geological basement (Basement), and downstream meanders without proximal geological basement (Non-basement) for the years of 1988 to 2023. Error bars represent standard deviation.

*δ*_*n*_ is then divided by the change in time (Δ*t*) to reach an annual migration rate (*ζ*_*n*_)^54^. *ζ*_*n*_ is averaged across the 100 data points of the meander bend to calculate the mean annual meander bend migration rate (*ζ*/m y^−1^) and then adjusted for the bend length to determine the dimensionless mean bend normalised annual meander migration rate (*ζ*_*B*_/y^−1^). *ζ* is used to evaluate the relationships between migration rate and different channel parameters (Fig. 3), while *ζ*_*B*_ is used to evaluate alongstream trends in migration rate while removing the intrinsic influence exerted by meander bend length (Figs. 2, 4).

3 $$S = \frac{channel\;length}{{downvalley\;length}}$$

Sinuosity (*S*) is calculated as the ratio of the length of a channel and the downvalley length^63^.

4 $$C = \left[ {\frac{dx}{{ds}}\frac{{d^{2} y}}{{ds^{2} }} - \frac{dy}{{ds}}\frac{{d^{2} x}}{{ds^{2} }}} \right] \cdot \left[ {\left( {\frac{dx}{{ds}}} \right)^{2} + \left( {\frac{dy}{{ds}}} \right)^{2} } \right]^{ - 3/2}$$

Curvature (*C*) is calculated using an established methodology based on the first and second-order derivatives of the Cartesian co-ordinates, *x* and *y*, and the intrinsic co-ordinate *s*, at each of the 100 equidistant data points along each meander bend^6,54^.

5 $$Skew = \frac{a - b}{{bend\;wavelength}}$$

Prior studies have calculated skewness as the angle between the midpoint and apex of a meander bend, with reference to a baseline^9^. Asymmetry has also been calculated in the past, with reference to the curvature maximum and points of inflection^65^. However, it is possible for meanders to have more than one apex of curvature maxima. In this study, skewness is defined, with reference to the 100 equidistant points of each meander bend, as the ratio of the distance between the downstream inflection point and the midpoint (*a*) and the distance between the upstream inflection point and the midpoint (*b*), in proportion to the bend wavelength. Negative skewness represents a downstream skewed meander bend, while upstream skewed bends exhibit positive skewness.

## Results

The studied stretch of meanders (1 to 24C) is presented in Fig. 1, while Supplementary Fig. S1 displays aerial images of selected meander bends. There are two confluences along this stretch of the river, a minor confluence between meanders 15B and 15C (Fig. 1a), and a major confluence just south of meander 17 (Figs. 1a, S1). An abandoned channel is present to the east of meanders 1 to 16 and numerous remnant oxbow lakes are visible along this stretch of the river (Figs. 1a, S1), while the downstream stretch of studied meanders, from meander 15C to 24C, are bounded by geological basement rock^48^ directly to their east (Figs. 1a, S1). The approximate tidal limit^49^ lies along meander 14 (Figs. 1a, S1). From the Landsat imagery, a downstream trend of increasing meander bend length and decreasing channel sinuosity is observable (Fig. 1a). Meanders bounded by oxbow lakes (Fig. S1) and geological basement (Fig. S1) are elongated and less curved than those not associated with resistant substrates (Fig. S1). The tight meander at the downstream end of the study area experienced rapid morphological changes after the chute cut-off event in 2014 (Fig. S1).

### Meander migration rate and channel morphodynamics

Meander migration rate was calculated on an annual basis for the 28 meanders identified during the period of 1988 to 2014, the 24 meanders identified between 2015 and 2016, and the 23 meanders identified between 2017 and 2023 (see Supplementary Table S1). Notably, the peak migration rate at meander 18 of 0.38 *ζ*_*B*_ (Fig. 2a) lies directly after the major confluence present after meander 17 (Fig. 1a). This confluence marks a shift in the magnitude of migration rate in comparison with the upstream portion of the studied stretch. The peak migration rate of 0.37 *ζ*_*B*_ at meanders 24B and 24C also exists south of this confluence (Fig. 2a). Meander 24B extends uninhibited to the south-west into agricultural land, while meander 24C rotates to the north-west, culminating in the anthropogenically initiated cut-off between bends 24A and 24C in 2014 (Figs. 1f,g, S1). Peaks of migration rate in the upstream stretch of meanders lie at meanders 6B and 14 (Fig. 2a); these meanders are also both involved in cut-off events. Meander 6B extends to the north-west, before it is eventually cut-off in 2014 (Fig. 1b,c). Meander 14 translates to the south, meeting meander 16 before achieving cut-off in 2017 (Fig. 1d,e).

Migration rate decreases with increasing curvature (Fig. 3a). Cases of extreme migration rates greater than 1500 *ζ* occurred only for meanders with curvatures less than 0.0010, while migration rates for meanders with curvatures greater than 0.0017 did not exceed 700 *ζ* (Fig. 3a). A similar, yet less distinctive, relationship exists between migration rate and sinuosity, where extreme cases of meander migration rate greater than 1500 *ζ* occurred when meander bend sinuosity did not exceed 1.4 (Fig. 3b). However, in some cases meander bends with sinuosities of up to almost 4 migrated at a rate of over 1000 *ζ* (Fig. 3b).

Meanders downstream of meander 13 exhibited less downstream skew than meanders further upstream (Fig. 2d). Only meanders with negligible skew in either the upstream or downstream direction (+ 0.1 > Skew >  − 0.1) exhibited migration rates greater than 1500 *ζ* (Fig. 3c), indicating that at the peak of their migration rate, rapidly migrating meanders were almost perfectly symmetrical in nature. However, it is difficult to identify a general relationship between skew direction and migration rate as, outside of the extreme cases of migration, meanders of varying positive and negative skew migrated at similar rates regardless of their skew direction. A more straightforward relationship is observable between meander bend half-wavelength and migration rate, whereby the potential magnitude of the migration rate increases in line with the increasing bend half-wavelength (Fig. 3d). A complex relationship is identified between sinuosity and skewness, whereby sinuosity increases in line with increasing skewness up to a skewness of approximately +/− 0.3 (Fig. 3e). The lack of data points for extremely skewed meander bends makes it difficult to define this relationship above or below a skewness of +/− 0.25 (Fig. 3e). Fitting formulae of the polynomial regression lines plotted in Fig. 3 are provided in Supplementary Table S2.

### Environmental influence on meander migration rate and channel morphodynamics

Figure 4 presents an analysis of migration rate for meanders exhibiting certain locally distinct characteristics. Eastward facing meanders migrate at a slower rate of 0.21 *ζ*_*B*_ compared to their westward facing counterparts, which migrated at an average of 0.25 *ζ*_*B*_ (Fig. 4a). Likewise, the downstream stretch of meanders, from meander 18 to 24C, migrating towards a prominent geological basement also exhibit a lower mean rate of migration of 0.25 *ζ*_*B*_ in contrast to those meanders without adjacent basement rock, which migrated on average at 0.28 *ζ*_*B*_ (Fig. 4a). Despite this, when compared to the average migration rate for all eastward facing meanders of 0.21 *ζ*_*B*_, meanders facing geological basement actually migrated at a comparably faster rate. Interestingly, meanders facing remnant oxbow lakes migrated at a faster rate of 0.25 *ζ*_*B*_ compared to those meanders without oxbow lakes which migrated at 0.22 *ζ*_*B*_ (Fig. 4a). Meanders extending towards a residual oxbow lake exhibited a lower mean curvature of 0.0012 (Fig. 4b), but higher mean sinuosity of 1.60 compared to other meanders (Fig. 4c). Downstream meanders migrating towards a geological basement were notably longer, with a mean length of 2329.80 m, when compared to those downstream meanders which could extend uninhibited by basement rock, which had a mean length of 2037.82 m (Fig. 4e).

### Precipitation in the Sittaung River drainage basin during peak ENSO years

**Figure 5:**
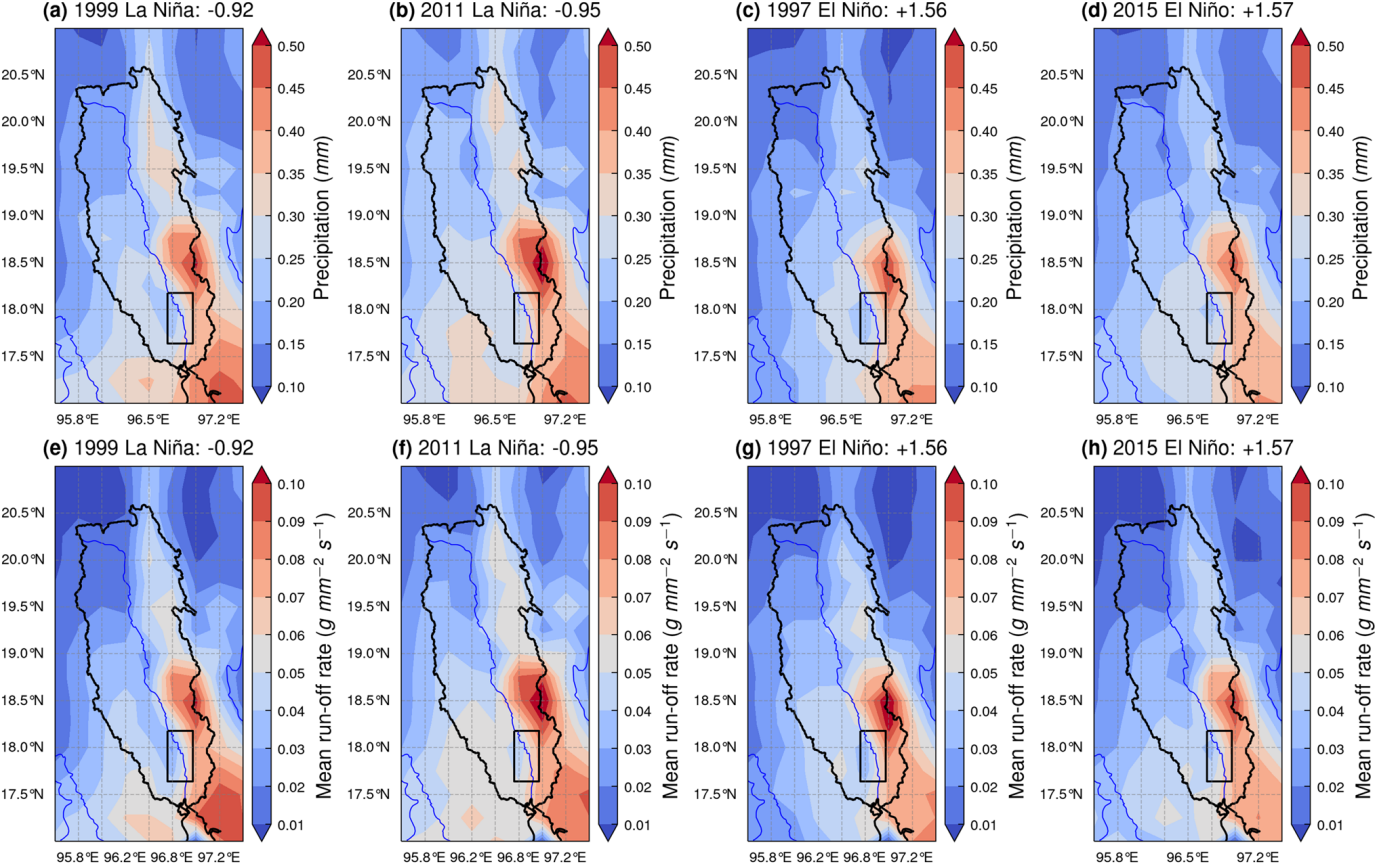
Maps showing ECMWF Reanalysis v5 (ERA5) (**a**–**d**) mean annual precipitation (mm) and (**e**–**h**) mean annual run-off rate (g mm^−2^ s^−1^) for peak El Niño and La Niña years (drainage basin boundary derived from the HydroBASINS dataset of the HydroSHEDS project^66^).

Figure 5 illustrates spatial trends in precipitation and run-off rate in the Sittaung River drainage basin^66^ during peak ENSO years. During the peak summertime La Niña years of 1999 (Niño 3.4: − 0.92) (Fig. 5a) and 2010 (Niño 3.4: − 0.95) (Fig. 5b), mean annual precipitation in the northern half of the basin exceeds that of the peak summertime El Niño years of 1997 (Niño 3.4: + 1.56) (Fig. 5c) and 2015 (Niño 3.4: + 1.57) (Fig. 5d). This is most notable along the north-eastern side of the basin, where there are regions of precipitation north of 19 degrees north reaching 0.30 mm during peak La Niña years (Fig. 5a,b), while these regions of higher precipitation are absent during peak El Niño years (Fig. 5c,d). During the peak La Niña years, the region of high annual mean precipitation to the northern side of the study area extends further to the north and east, reaching 0.30 mm and 0.35 mm at 19 degrees north and 96.75 degrees east respectively (Fig. 5a,b), compared to only 0.25 mm and 0.30 mm in the same locations during El Niño years (Fig. 5c,d). Spatial trends in mean run-off rate between El Niño and La Niña years broadly align with those exhibited for mean precipitation, with the region of high run-off rates reaching 0.06 g mm^−2^ s^−1^ extending toward the north-east of the drainage basin present in peak La Niña years (Fig. 5e,f), but absent during peak El Niño years (Fig. 5g,h).

**Figure 6:**
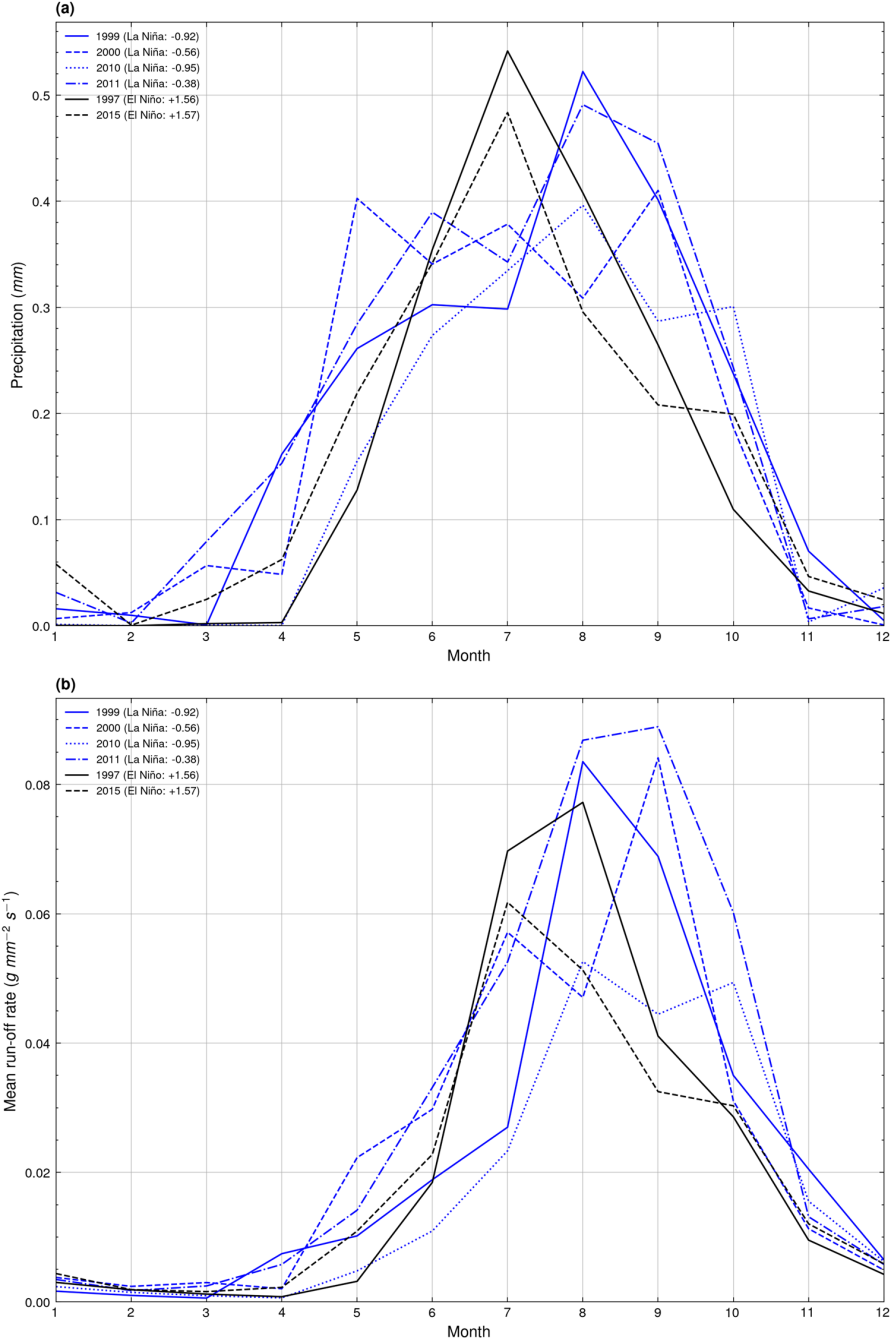
Graphs showing ECMWF Reanalysis v5 (ERA5) (**a**) monthly spatial mean precipitation (mm) and (**b**) monthly spatial mean run-off rate (g mm^−2^ s^−1^) for peak El Niño and La Niña years (spatial extent: 95.75° E 19.00° N 98.00° E 20.50° N).

Figure 6 illustrates ERA5 monthly mean precipitation and run-off rate^53^ based on the spatial extent of the drainage basin shown in Fig. 5. Between the peak El Niño and La Niña years, a distinct contrast in the monthly precipitation pattern is visible. The El Niño years of 1997 and 2015 exhibit a solitary peak monthly mean precipitation in July of 0.54 mm and 0.48 mm respectively, with figures continually decreasing either side of this peak (Fig. 6a). This pattern of monthly precipitation differs from that illustrated for the peak La Niña years of 1999, 2000, 2010, and 2011, which each exhibit multiple peaks of precipitation from the month of June, to as late as October (Fig. 6a). The monthly trend in mean run-off rate during peak El Niño and La Niña years lags behind that of the pattern in mean monthly precipitation, with El Niño years exhibiting later and less clearly defined peaks in mean run-off rate (Fig. 6b). La Niño years still exhibited multiple peaks of monthly mean run-off rate on occasion, in the years of 2000 and 2010, however the period of high monthly mean run-off rate was more concentrated towards the latter half of the year, between the months of July to October (Fig. 6b), compared to the more extensive period of high monthly precipitation (Fig. 6a).

### Meander migration rate versus Niño 3.4 index

**Figure 7:**
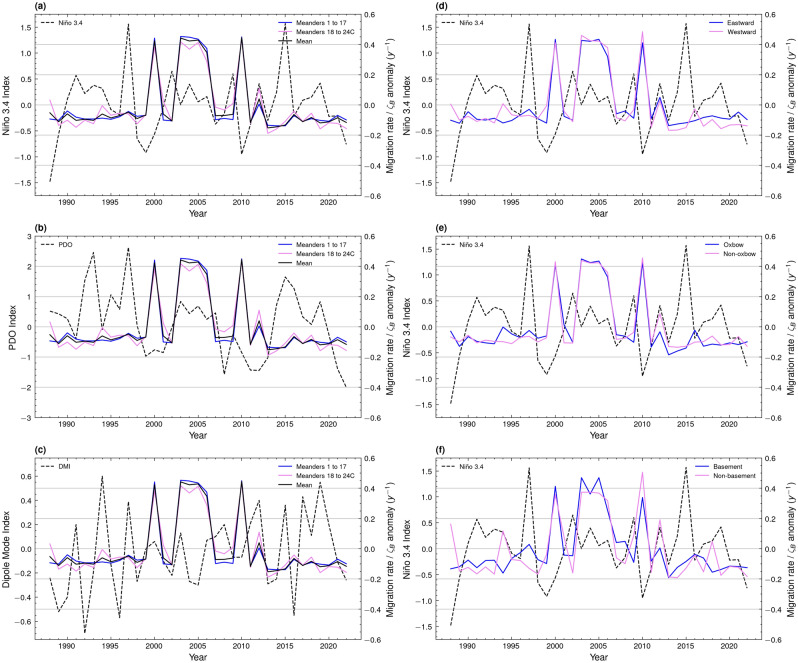
Graphs showing the relationships between annual migration rate anomaly (*y*^−1^) and summertime (June, July, August) (**a**) Niño 3.4 Index, (**b**) PDO Index, and (**c**) Dipole Mode Index (DMI). The relationship between annual migration rate anomaly (y^−1^) and Niño 3.4 Index is presented for (**d**) eastward (Eastward) and westward (Westward) facing meanders, (**e**) meanders with (Oxbow) and without (Non-oxbow) proximal oxbow lakes, and (**f**) downstream meanders with (Basement) and without (Non-basement) proximal geological basement.

The time series of the summertime (June, July, and August) Niño 3.4 Index and the annual migration rate anomaly presented in Fig. 7a shows the relationship between ENSO and the evolution of the Sittaung River. Major peaks of El Niño events exist in 1997 (+ 1.56) and 2015 (+ 1.57), while minor peaks occurred in 2002 (+ 0.65) and 2009 (+ 0.60) (Fig. 7a). The periods of 1990 to 1994 (mean + 0.32) and 2017 to 2019 (mean + 0.22) are also dominated by a continuous El Niño signal (Fig. 7a). A continuous strong La Niña signal is present from 1998 to 2000 (− 0.65, − 0.92, − 0.56), while the lowest La Niña index of − 0.95 is present in 2010 (Fig. 7a). For the period of 2003 to 2008 (mean + 0.01), there is no strong signal of El Niño or La Niña, indicating negligible forcing from ENSO during these years (Fig. 7a).

Anomalies of migration rate were calculated on an annual basis with respect to the mean of the entire migration rate time series from 1988 to 2023. El Niño events exhibited negligible forcing on the migration rate of the Sittaung River, with low-to-average migration rates recorded during El Niño periods (Fig. 7a). Major El Niño events in 1997 and 2015 resulted in migration rate anomalies of − 0.048 *ζ*_*B*_ and − 0.133 *ζ*_*B*_ respectively, while minor events in 2002 and 2009 resulted in anomalies of − 0.106 *ζ*_*B*_ and − 0.062 *ζ*_*B*_ (Fig. 7a). The major La Niña event in 2010 coincided with a peak migration rate anomaly of + 0.445 *ζ*_*B,*_ while a peak in migration rate anomaly of + 0.424 *ζ*_*B*_ also occurred in 2000, during the period of dominant La Niña signal (Fig. 7a).

Figure 7 also illustrates the sensitivity of local environmental factors, such as meander orientation, oxbow lake presence, and geological basement, to climate forcing by El Niño and La Niña. Eastward and westward facing meanders (Fig. 7d), and meanders with or without a residual oxbow lake (Fig. 7e) exhibited a similar migration rate anomaly time series to the overall mean, yet the time series for meanders downstream of meander 17 with or without proximal geological basement (Fig. 7f) fluctuates about the mean migration rate time series. Despite exhibiting similar anomalies of migration rate during the 2000 La Niña event, during the major La Niña event in 2010, meanders without proximal basement rock recorded a migration rate anomaly of + 0.51 *ζ*_*B*_ compared to that of + 0.34 *ζ*_*B*_ for meanders bounded by geological basement (Fig. 7f).

**Figure 8:**
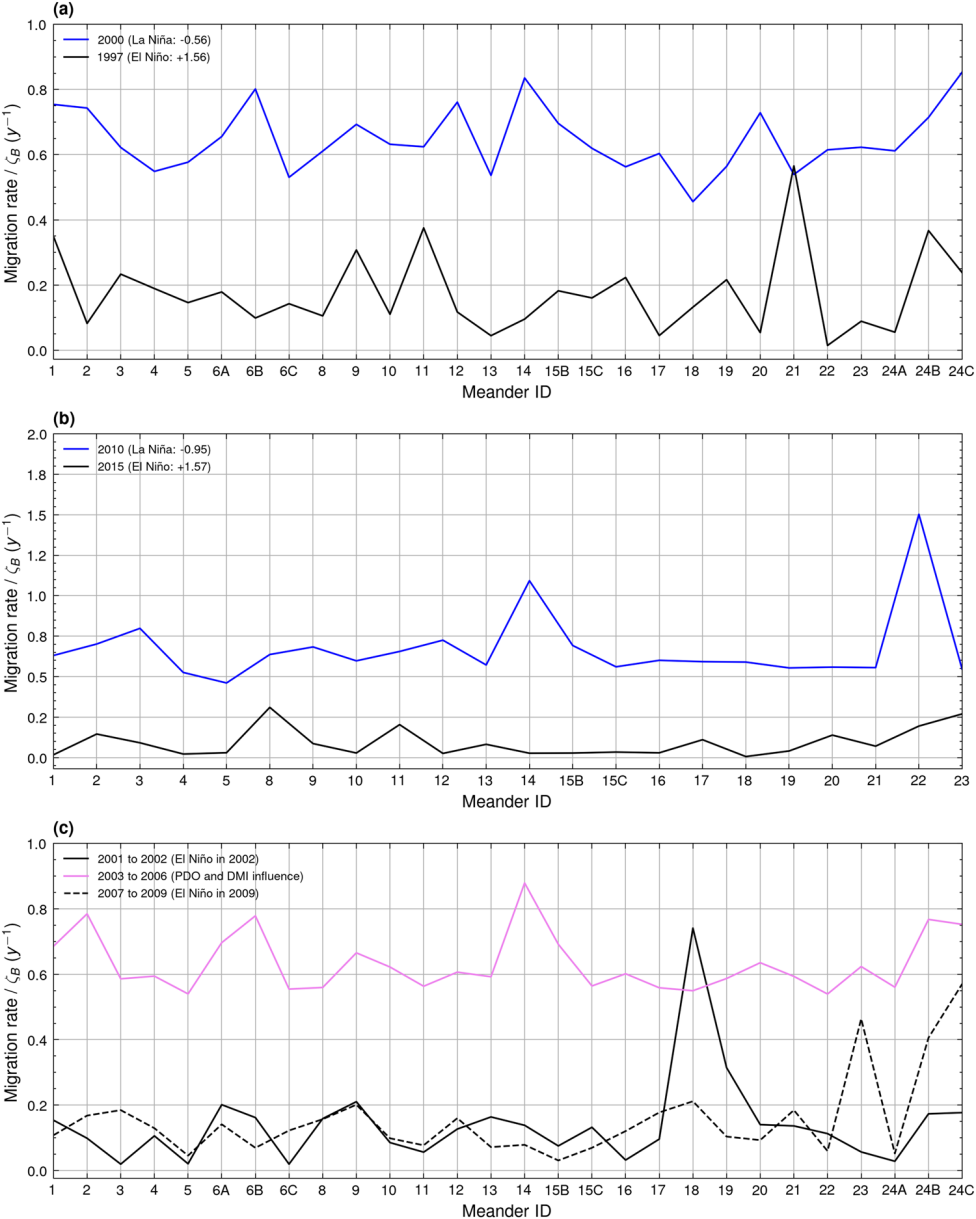
Migration rate (y^−1^) for meanders present during periods of El Niño, La Niña, and PDO and DMI influence. There is a significant difference at the 95% confidence interval between (**a**) 1997 and 2000 (t value: − 15.47, *p* value: 6.08e^−15^, df: 27), (**b**) 2015 and 2010 (t value: − 13.13, *p* value: 3.62e^−12^, df: 23), and (**c**) the mean of the years 2001, 2002, 2007, 2008, and 2009 and the mean of the years 2003, 2004, 2005, and 2006 (t value: − 21.40, *p* value: 1.83e^−18^, df: 27).

When compared to the migration rate recorded during the El Niño periods of 1997 and 2015, the La Niña periods of 2000 and 2010 exhibited a greater mean migration rate for each meander bend along the stretch (Fig. 8a,b). There was a significant difference between the migration rates of these periods at the 95% confidence level (Fig. 8a,b).

### Meander migration rate versus PDO and IOD indices

In the absence of a strong summertime ENSO signal, there is the possibility that other teleconnections may influence the intensity of monsoon rains delivered to the Sittaung River drainage basin. For the period of 2003 to 2006, despite the lack of La Niño forcing, the mean migration rate anomaly lies at + 0.410 *ζ*_*B*_, peaking at + 0.442 *ζ*_*B*_ in 2003 at a summertime ENSO Index of 0.00 (Fig. 7a). During the same period, there was a continuously positive summertime PDO Index, averaging + 0.56, peaking in 2003 at + 0.84 in unison with the peak in migration rate (Fig. 7b). A trough in the summertime Dipole Mode Index (DMI), representative of the strength in the IOD, averaging − 0.29 from 2004 to 2005 also occurred during this period of sustained peak migration rate (Fig. 7b). There was a significant difference at the 95% confidence level between the mean migration rate of the 2003 to 2006 period compared to the combined mean of the immediately preceding period of 2001 to 2002 and following period of 2007 to 2009 (Fig. 8c).

## Discussion

### Climate control on meander migration rate

Climate oscillations are a driving force behind the annual migration of the meander bends of the Sittaung River. Their control of the position, duration, and intensity of monsoon rains can influence the fluvial discharge and sediment input into the river^25–27^, either accelerating or inhibiting the rate of migration^19,20^. As illustrated in Figs. 5 and 6, there is both a spatial and temporal control on precipitation and run-off rate which is exerted by El Niño and La Niña. During La Niña years, the region of high precipitation within the Sittaung River drainage basin is extended further northwards than that during El Niño years (Fig. 5), while high monthly mean precipitation is maintained throughout the monsoon period from June to October, compared to the single peak of precipitation in June exhibited during El Niño years (Fig. 6a). These factors are likely to have led to the peaks in migration rate during the La Niña years of 2000 and 2010 (Fig. 7a).

From 1990 to 1997 and 2011 to 2021, the summertime monsoon is controlled in the majority by El Niño conditions (Fig. 7a), whereby the Walker circulation shifts east^30^, bringing dry conditions to Myanmar^33^. These dry conditions are likely to have caused the relatively stable migration rate observed during these periods (Fig. 7a), as it is probable that the river lacked the requisite fluvial discharge or sediment input to fuel its migration. The years of 1998 to 2000 and the year of 2010 represent the strongest La Niña forcing (Fig. 7a), during which the Walker circulation shifts west^30^, leading to intense monsoon rainfall in Myanmar^31,32^. These strong monsoon conditions have likely induced peaks of migration rate in both 2000 and 2010 (Fig. 7a). However, it should be noted that it is unusual that the peak migration rate in 2000 falls a year after the peak La Niña index in 1999, and the positive migration rate anomaly is not continuous throughout 1998 to 2000, rather only peaking in 2000. A potential factor driving the peaks of migration rate in 2000 and 2010 is that multiple peaks of both monthly mean precipitation (Fig. 6a) and monthly mean run-off rate (Fig. 6b) existed throughout these La Niña years, indicating a long and intense monsoon season, capable of driving meander migration over the span of multiple months.

ENSO cannot be attributed as the sole climatic driver influencing the Sittaung River’s morphological evolution. The years of 1988 (− 1.48) and 2022 (− 0.76) both exhibit a strong La Niña signal, yet there is no notable forcing in the time series of meander migration rate (Fig. 7a). The lack of data availability pre-1988 and post-2023 means that it is difficult to view cyclical ENSO patterns which could help establish the reason for this apparent lack of climatic forcing. In the absence of ENSO forcing during the period of 2003 to 2008, the signals of the PDO and IOD offer insight into what may have caused the continuous peak of migration rate from 2003 to 2006. During years of weak ENSO forcing, the positive phase of the PDO has been shown to lead to wetter conditions^34,35^. This PDO forcing, combined with the forcing of the negative IOD during this period, is likely to have contributed to the high rate of meander migration during these years.

### Factors obscuring climate forcing

Climate oscillations drive the morphodynamics of the Sittaung River through their control of monsoon precipitation and in turn, fluvial discharge^25–27^. As such, their influence on the morphological change of the Sittaung is purely fluvially driven. Non-fluvial processes such as tidal modulation^49,50^, and anthropogenic influences such as mining^39^, agriculture, and channel construction have the potential to obscure the signal of teleconnections in the time series of meander migration. This is particularly prevalent for downstream meanders which are more likely to be modulated by tidal processes. The reduced sensitivity of downstream meander bends to climatic forcing can be examined by comparing the migration rate of meanders 1 to 17 with that of meanders 18 to 24C (Fig. 7a). During years of peak meander migration in 2000, 2003 to 2006, and 2010, the upstream stretch of meanders 1 to 17 recorded consistently higher positive migration rate anomalies (mean + 0.430 *ζ*_*B*_) compared to the downstream stretch of meanders 18 to 24C (mean + 0.377 *ζ*_*B*_), while during dry El Niño conditions meanders 18 to 24C exhibited visibly greater fluctuation in their annual migration rate compared to the more stable evolution of meanders 1 to 17 (Fig. 7a). This suggests that tidal modulation may be masking the climate forcing of these downstream meanders’ migration, weakening the influence of high fluvial discharge driven by monsoon rains^21–23^.

Local environmental conditions have the potential to both inhibit and accelerate the rate of meander migration, masking the signal of climate drivers. Environmental controls may dictate the prominent mode of meander migration, consequently influencing the rate of migration^8^. The presence of an abandoned channel to the east of meanders 1 to 16 (Fig. 1a) and the geological basement^48^ located to the east of meanders 15C to 23C may have impeded the extension of some eastward facing meanders in these regions, resulting in their notably lower rate of migration compared to their more freely migrating westward facing counterparts (Fig. 4a). The presence of metamorphic basement rock^48^ in particular appears to have had a direct influence on the mode of meander migration. While westward facing meanders migrated at a greater rate on average (Fig. 4a), meanders facing a geological basement migrated faster than the overall mean of eastward facing meanders (Fig. 4a), indicating the inhibition of extension and promotion of migration by way of more rapid seaward translation.

There are other factors that may have influenced the sensitivity of meanders 18 to 24C to climate forcing. The fluvial input of water and sediment^15^ at the confluence south of meander 17 may have fuelled meander migration downstream of this confluence (Fig. 1a). However, anthropogenic activities, rather than climatic forcing, may control the seasonal fluctuations of these fluvial inputs into the river system. Over the course of the study period, the Shwegyin Dam and reservoir were constructed to the north-east of the study area (Fig. 1a). Periods of storing or releasing water from this reservoir^43,44^ could have impacted the extent of fluvial inputs at the confluence south of meander 17. The input of sediment at this confluence from upstream mining activities^39^ may have also been a contributing factor to the increased variability in the migration rate for meanders 18 to 24C.

### Channel morphodynamic relationships

The studied meanders of the Sittaung River migrated at an average rate of 349 *ζ* (m y^−1^). This rapid rate of migration emphasises the relative dynamism of these meandering channels when compared with those of other fluvial-tidal systems. Further downstream, the bank erosion of the Sittaung Estuary peaked at 3000 *ζ*^50^. In contrast, the channels of other major rivers, such as those of the Irrawaddy, Indus, and Ganges, migrated at rates of up to 60 *ζ*^20^, while tidal meanders studied in the Venice Lagoon^54^ and New Jersey Wetlands^67^ migrated at rates of less than 3 *ζ*, frequently lower than 1 *ζ*. As such, the dynamic channels of the Sittaung are ideal for the study of the morphodynamic response and sensitivity of meanders to forcing from short-term climate fluctuations.

The eastward extension of meander bends south of meander 15C is likely to have been controlled by the basement rock to the east of this downstream stretch of the river^48^. Over the course of the study period, meanders 15C, 17, 19, 21, and 22 (Fig. 1a), despite facing eastward, exhibited negligible eastward extension. These meanders each had low mean curvatures (Fig. 2b), possibly a result of downstream translation, rather than extension, being their most prominent mode of migration. Through forcing these meanders to migrate by means of translation rather than extension, a resultant effect appears to be their longer half-wavelength compared to their neighbouring meander bends (Fig. 2e), suggesting that the downstream end of these meanders translated at a greater rate than their upstream end. This phenomenon is similar to the elongate bends that are observable for some bedrock meanders^12^. Conversely, all the meanders of peak curvature, meanders 6A, 7A, 12, and 15A (Fig. 2b), while also facing to the east, were short meanders (Fig. 2e) found north of meander 15C (Fig. 1a). As such, these meanders were able to extend eastward uninhibited by basement rock, resulting in their comparatively higher mean curvatures (Fig. 2b).

Meander migration has been shown to sharply decrease beyond peak curvature^5,6^, a relationship with which the results of this study concur (Fig. 3a). Yet, this relationship is inherently associated with the mode by which a meander bend migrates. If a meander bend were to migrate solely by extension, its points of inflection would remain static while its maximum curvature would increase. Likewise, a meander solely migrating through translation would migrate downstream, while its maximum curvature would remain unchanged. In the case of this study, among all the eastward facing meanders (0.21 *ζ*_*B*_), meanders facing basement rock actually exhibited a higher rate of migration (0.25 *ζ*_*B*_) (Fig. 4a). This suggests that, while the eastward extension of this set of meanders was inhibited by the basement rock, their seaward translation may have resulted in overall higher rates of migration^8^. Further upstream, eastward facing meanders which migrated more prominently through extension likely reached peak curvature at a faster rate, inhibiting the rate of their continued migration.

In a completely ebb-dominated system, meanders should be predominately downstream skewed, in line with the flow of ebb currents^65^. However, in a tide-dominated estuary setting such as the Sittaung, despite ebb-dominance upstream of the bedload convergence point, tidal modulation can affect channel morphodynamics up to the tidal limit^68^. In the case of this study, meanders downstream of meander 13 exhibited less downstream skew than meanders upstream of that point (Fig. 2d). This indicates that despite strong fluvial dominance during the monsoon season, tidal modulation during the dry season may have reduced the extent of these meanders’ downstream skew.

### Advantages and limitations of a satellite-based study approach

The use of satellite data in this study provided advantages over a traditional observation-based approach. The ERA5 dataset supplied both a continual temporal and spatial record of precipitation and run-off data from which a time series could be extracted; in contrast with observational data which would provide point data at only specific locations, during varying time-intervals, and collected using a range of unverifiable methodologies. In the absence of a reliable spatio–temporal observational dataset, using these variables as a proxy for fluvial discharge enables the study of multi-decadal climate control of meander migration rate. Despite the limited spatial resolution of approximately 30 km by 30 km, through the calculation of a spatial mean, climatic parameters within the Sittaung River drainage basin can be illustrated and interpreted. The Landsat dataset used in this study also has a limited spatial resolution of 30 m by 30 m, yet by approximating the centreline from the channel banks and smoothing the resultant vector, calculations on a more precise scale can be reliably performed.

The ERA5 dataset has been used in studies to evaluate precipitation in East Asia, exceeding other satellite-based datasets in terms of its estimation accuracy, particularly in non-urbanised areas during monsoon season, despite its relatively large estimation bias^69–71^. The dataset effectively illustrates regional trends in precipitation, however has limitations in replicating maximums of observed precipitation and shows greater effectiveness in modelling extra-tropical regions compared to the sub-tropics^72^.

The use of precipitation and run-off data as a proxy for fluvial discharge enables the study of climate forcing on the Sittaung River’s channel morphodynamics, despite the lack of reliable local fluvial discharge measurements. This is particularly valuable in the case of a country such as Myanmar, which is presently engaged in a conflict, restricting the collection of in-situ measurements.

GIS automated centreline generation provides an accessible and repeatable method from which channel morphodynamics can be analysed. Compared to traditional methods of on-site data collection, or the geo-referencing of aerial or LiDAR imagery, this automated process presents a reliable alternative that eliminates many of the errors associated with the manual collection and manipulation of data. Additionally, rather than using a global thresholding method, such as Otsu’s method^56–60^, using a local adaptive thresholding method enables improved segmentation of the Landsat image along the entire stretch of the river channel.

This approach is not without its limitations. Post-2003, Landsat-7 data is not suitable for use in the thresholding algorithm due to damage to the Landsat-7 Scan Line Corrector, which resulted in major data gaps^52^. As such, data used from 2003 onwards was collected from Landsat-5 until the introduction of Landsat-8 in 2013. Cloud cover also presents difficulties in Landsat image selection. When cloud is present in a Landsat image, it is unusable for thresholding as the cloud acts as a mask over the river channel, preventing the channel from being defined by the thresholding algorithm.

## Conclusions

In this study, the time series of meander bend migration rate is interpreted with reference to channel morphodynamics and the climate oscillation signals of the ENSO, PDO, and IOD. Meander bend migration rate peaked south of a confluence where the discharge and sediment input into the river system at this confluence fuelled migration. Meander bend curvature was controlled by basement rock and high meander bend sinuosity acted as an indicator of cut-off events. There is significant climatic forcing from ENSO, which drives the migration of the meander bends of the Sittaung River. In years of El Niño conditions, a low-to-average rate of migration is expected, while under strong La Niña conditions, the extended monsoon season can lead to peaks of migration rate. The extent of forcing exerted by ENSO is affected by local environmental conditions, notably the presence of proximal geological basement which can inhibit the extension of meander bends, forcing them to migrate primarily by translation. During periods of consistently weak ENSO forcing, the PDO and to a lesser extent the IOD may dictate the intensity of monsoon precipitation and therefore the rate of fluvially driven meander migration. However, a downstream decrease in the sensitivity of climate forcing was identified, where tidal modulation and anthropogenic influences may have obscured climate signals in the annual meander migration rate time series.

### Supplementary Information

Supplementary Information.

## Data Availability

The datasets generated during and/or analysed during the current study are available from the corresponding author on reasonable request.
