# Supplementary material for: Climate control on the channel morphodynamics of the Sittaung River, Myanmar
Source: Sci Rep. 2024 Mar 29;14:7524. doi: 10.1038/s41598-024-58198-1 (PMC10980759; doi:10.1038/s41598-024-58198-1)
Supplement: Supplementary file 1 — Supplementary Information. [file 41598_2024_58198_MOESM1_ESM.pdf]

Climate control on the channel morphodynamics of the Sittaung River, Myanmar: Supplementary Information

Luke Stefan Bisson*, **Kyungsik Choi**

School of Earth and Environmental Sciences and Research Institute of Oceanography, Seoul National University, Seoul, Republic of Korea

* Corresponding author: Luke Stefan Bisson (lukebisson@snu.ac.kr)

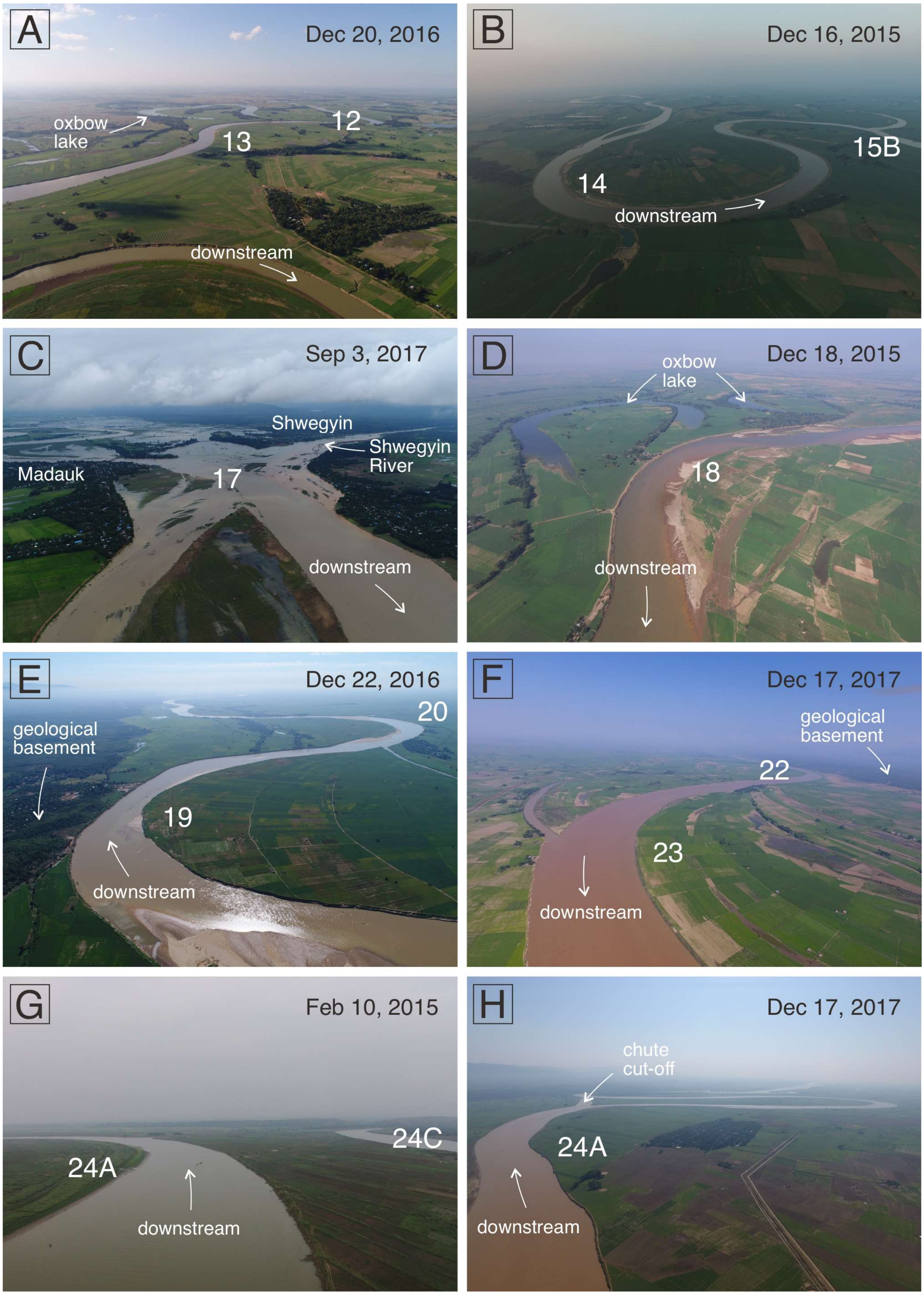

Supplementary Figure S1. Aerial photographs showing the meanders of the Sittaung River.

Numbers denote meander ID as shown in Figure 1.

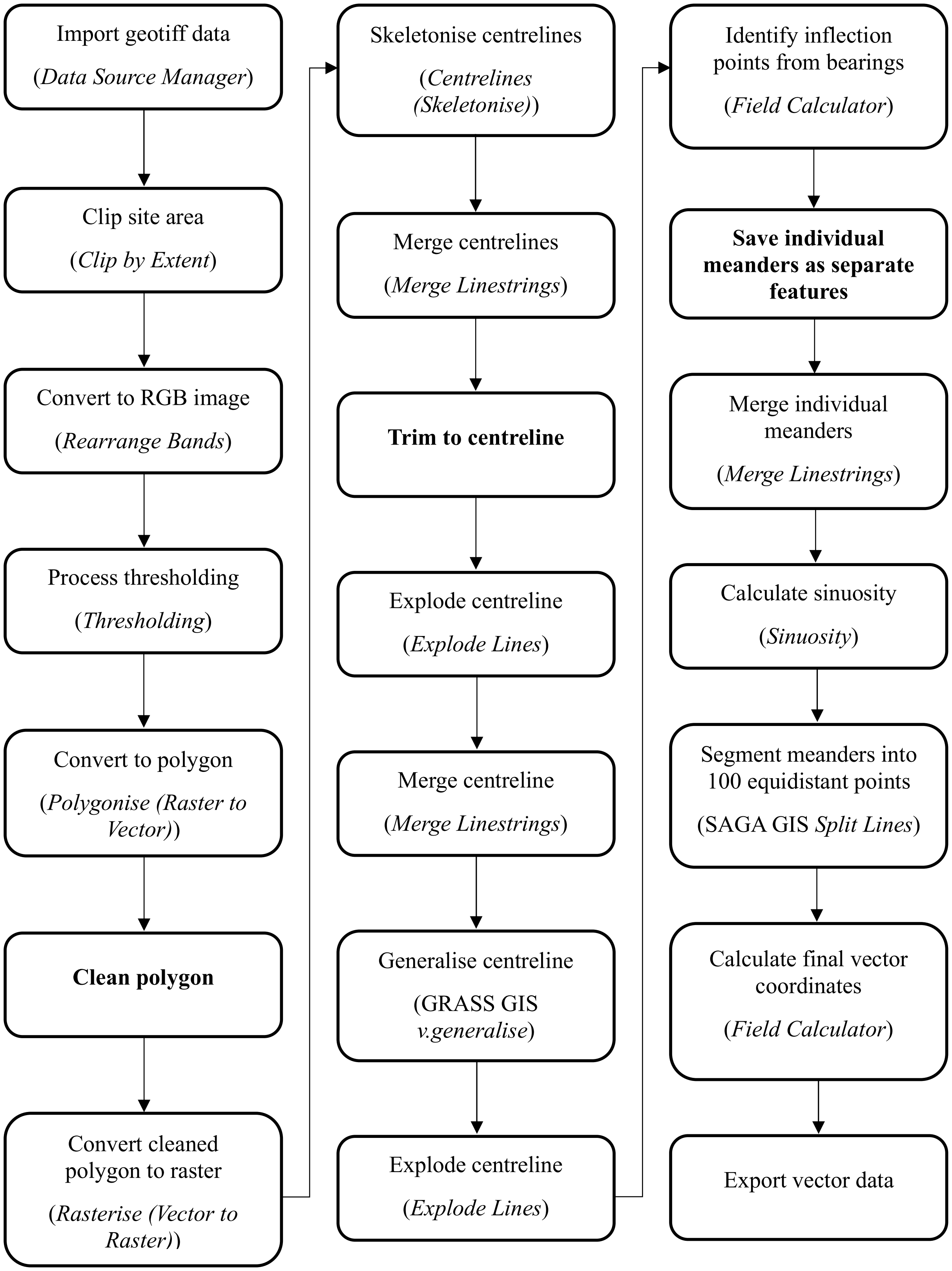

Supplementary Figure S2. Flowchart showing data processing procedures performed in QGIS. QGIS functions are italicised, while manual processes are in bold.

Supplementary Table S1. Mean meander bend parameters for the years of 1988 to 2023

Meander ID	Migration rate / ζ ($m\ y^{-1}$)	Curvature / C	Sinuosity / S	Skewness	Bend half-wavelength (m)
1	352.9492	0.0011	1.7225	0.0412	1743.8957
2	288.9702	0.0013	2.1847	0.0843	1213.3002
3	292.7321	0.0011	1.3622	-0.0608	1507.4106
4	243.0911	0.0009	1.0666	-0.0025	1311.4549
5	282.7699	0.0006	1.0822	0.0022	1883.3494
6A	197.1535	0.0019	1.8146	0.0021	776.7066
6B	403.4582	0.0015	2.0564	-0.1029	1242.1654
6C	313.7077	0.0009	1.1043	-0.0040	1414.1909
7A	135.2888	0.0022	1.1474	0.0302	702.0946
7B	123.0264	0.0014	1.0529	0.0077	814.4895
8	253.4560	0.0016	1.3615	0.0554	1166.1250
9	235.8517	0.0017	1.6556	-0.0714	1001.2728
10	264.8964	0.0011	1.4551	-0.0419	1735.2153
11	201.9720	0.0016	1.3702	-0.0413	930.5869
12	160.0784	0.0021	1.3475	-0.0252	752.3390
13	255.9410	0.0009	1.1369	0.0009	1453.3907
14	327.5600	0.0014	2.2434	-0.0535	1160.8892
15A	100.6124	0.0023	1.3283	0.0325	710.9356
15B	327.5375	0.0016	1.5518	0.0114	1295.4203
15C	341.0262	0.0010	1.2025	0.0112	1559.1524
16	214.1731	0.0015	1.4324	0.0203	1216.9821
17	234.1230	0.0011	1.1363	-0.0038	1396.8850
18	543.1561	0.0011	1.0982	0.0084	1304.7822
19	349.8262	0.0010	1.2944	0.0258	2021.8452
20	473.9258	0.0009	1.2777	-0.0460	2152.2776
21	525.4413	0.0007	1.0982	0.0018	2301.9189
22	724.0203	0.0005	1.0732	-0.0054	3780.2885
23	689.8478	0.0007	1.1890	-0.0078	2827.9272
24A	515.0437	0.0007	1.2222	-0.0117	2573.8789
24B	491.0810	0.0012	2.0398	0.0515	1330.2372
24C	464.6266	0.0015	1.7460	-0.0517	1215.1593

Supplementary Table S1. Table showing the average alongstream meander bend migration rate ($m\ y^{-1}$), curvature, sinuosity, skewness, and bend half-wavelength (m) for the years of 1988 to 2023.

Supplementary Table S2. Fitting formulae of Fig. 3 regression plots	
Regression plot	Formulae
Migration rate ($m\ y^{-1}$) and curvature	$\zeta = -7.2130e^{+10} + (4.4703e^{+8})C - 982680.8571C^2 + 971.2769C^3$
Migration rate ($m\ y^{-1}$) and sinuosity	$\zeta = -42.8731 + 392.2823S - 967.2746S^2 + 1024.0718S^3$
Migration rate ($m\ y^{-1}$) and skewness	$\zeta = -1947.1453 + 2176.4196Skew + 254.3321Skew^2 + 336.3834Skew^3$
Migration rate ($m\ y^{-1}$) and bend half-wavelength (m)	$\zeta = -3.2448e^{-9} + (3.2594e^{-5})Length + 0.0839Length^2 + 140.7507Length^3$
Sinuosity and skewness	$S = 118.5830 - 159.7479Skew - 7.8555Skew^2 + 27.8557Skew^3 + 0.2677Skew^4 + 1.2940Skew^5$

Supplementary Table S2. Table showing fitting formulae for the regression plots of migration rate ($m\ y^{-1}$) and curvature, migration rate ($m\ y^{-1}$) and sinuosity, migration rate ($m\ y^{-1}$) and skewness, migration rate ($m\ y^{-1}$) and bend half-wavelength (m), and sinuosity and skewness.
